# Empiric guideline-recommended weight-based vancomycin dosing and nephrotoxicity rates in patients with methicillin-resistant *Staphylococcus aureus* bacteremia: a retrospective cohort study

**DOI:** 10.1186/2050-6511-14-12

**Published:** 2013-02-13

**Authors:** Ronald G Hall, Kathleen A Hazlewood, Sara D Brouse, Christopher A Giuliano, Krystal K Haase, Chistopher R Frei, Nicolas A Forcade, Todd Bell, Roger J Bedimo, Carlos A Alvarez

**Affiliations:** 1 Department of Pharmacy Practice, Texas Tech University Health Sciences Center, School of Pharmacy, Dallas, USA; 2 Department of Clinical Sciences, University of Texas Southwestern, Dallas, USA; 3 Department of Pharmacy Practice, Texas Tech University Health Sciences Center, School of Pharmacy, Amarillo, USA; 4 Division of Pharmacotherapy, University of Texas, Austin, USA; 5 Department of Internal Medicine, Texas Tech University Health Sciences Center, School of Medicine, Amarillo, USA; 6 Department of Internal Medicine, University of Texas Southwestern, Dallas, USA; 7 Current affiliation: University of Wyoming School of Pharmacy, Swedish Family Medicine Residency Program, Englewood, USA; 8 Current affiliation: University of Kentucky Healthcare, Lexington, USA; 9 Current affiliation: Wayne State University Eugene Applebaum College of Pharmacy and Health Sciences, Detroit, USA; 10 Current affiliation: Mission Regional Medical Center, Mission, USA

**Keywords:** Adverse events, Nephrotoxicity, Vancomycin, Dosing, Weight, Obesity, MRSA

## Abstract

**Background:**

Previous studies have established a correlation between vancomycin troughs and nephrotoxicity. However, data are currently lacking regarding the effect of guideline-recommended weight-based dosing on nephrotoxicity in methicillin-resistant *Staphylococcus aureus* bacteremia (MRSAB).

**Methods:**

Adults who were at least 18 years of age with methicillin-resistant *Staphylococcus aureus* bacteremia and received of empiric vancomycin therapy for at least 48 hours (01/07/2002 and 30/06/2008) were included in this multicenter, retrospective cohort study. The association between guideline-recommended, weight-based vancomycin dosing (at least 15 mg/kg/dose) and nephrotoxicity (increase in serum creatinine (SCr) by more than 0.5 mg/dl or at least a 50% increase from baseline on at least two consecutive laboratory tests) was evaluated. Potential independent associations were evaluated using a multivariable general linear mixed-effect model.

**Results:**

Overall, 23% of patients developed nephrotoxicity. Thirty-four percent of the 337 patients who met study criteria received weight-based dosing. The cohort was composed of 69% males with a median age of 55 years. The most common sources of MRSAB included skin/soft tissue (32%), catheter-related bloodstream bacteremia (20%), pulmonary (18%). Eighty-six percent of patients received twice daily dosing. Similar rates of nephrotoxicity were observed regardless of the receipt of guideline-recommended dosing (22% vs. 24%, OR 0.91 [95% CI 0.53-1.56]). This finding was confirmed in the multivariable analysis (OR 1.52 [95% CI 0.75-3.08]). Independent predictors of nephrotoxicity were (OR, 95% CI) vancomycin duration of greater than 15 days (3.36, 1.79-6.34), weight over 100 kg (2.74, 1.27-5.91), Pitt bacteremia score of 4 or greater (2.73, 1.29-5.79), vancomycin trough higher than 20 mcg/ml (2.36, 1.07-5.20), and age over 52 years (2.10, 1.08-4.08).

**Conclusions:**

Over one out of five patients in this study developed nephrotoxicity while receiving vancomycin for MRSAB. The receipt of guideline-recommended, weight-based vancomycin was not an independent risk factor for the development of nephrotoxicity.

## Background

Decreased vancomycin efficacy has been reported by several investigators for methicillin-resistant *Staphylococus aureus* (MRSA) isolates with a vancomycin MIC of 1 μg/ml or higher [1-3]. This has been accompanied by a trend of increased vancomycin minimum inhibitory concentrations (MIC) in MRSA isolates [4]. Experts in the field have responded in two ways. First, the Clinical and Laboratory Standards Institute lowered the susceptibility breakpoint for vancomycin versus *S. aureus*[5]. Second, several influential organizations endorsed a consensus review recommending higher vancomycin trough concentrations [6]. Weight-based dosing was recommended to achieve these new target vancomycin trough concentrations. The impact of these two changes on the safety profile of vancomycin is unknown. Recent studies suggest increased vancomycin trough concentrations are a risk factor for increased rates of nephrotoxicity [1,7-10]. However, no studies have yet evaluated the effect of guideline-recommended, weight-based vancomycin dosing on nephrotoxicity. We performed a multi-center retrospective cohort study to assess the risk of nephrotoxicity associated with guideline-recommended, weight-based vancomycin dosing.

## Methods

### Design

We conducted a multi-center, retrospective cohort study at three hospitals between July 2002 and June 2008. This retrospective cohort evaluated the association between the receipt of weight-based vancomycin dosing as recommended in the 2009 guideline and the development of nephrotoxicity in patients with MRSA bacteremia [6]. The development of nephrotoxicity during vancomycin therapy was the primary outcome of interest. All data were collected from the patient’s medical record at each study institution. The results of our evaluation of guideline-recommended, weight-based dosing and mortality can be found elsewhere [11].

### Setting

The three study hospitals were a 400 bed tertiary hospital, a 350 bed Veteran Affairs hospital, and a 600 bed university hospital. The requirement for informed consent was waived by each of the institutional review boards (IRBs) that approved the study (North Texas Veterans Health Care System, Texas Tech University Health Sciences Center, and University of Texas Health Science Center, San Antonio) due to the retrospective nature of the study and being deemed as minimal risk.

### Patients

All adults (18 years or older) admitted with MRSA bacteremia (identified by microbiological records) who received parenteral vancomycin for at least 48 hours were evaluated for study inclusion. Patients were excluded if at the time of the first vancomycin dose they were pregnant, had moderate-to-severe renal dysfunction (defined as a creatinine clearance (CrCl) ≤ 30 ml/min or receipt of dialysis), received vancomycin within the same hospital stay, or had a culture-proven MRSA infection within six months [12]. Patients with a CrCl ≤ 30 ml/min were excluded because these patients were considered more likely to require a dosing frequency adjustment to less than once daily dosing and the measure of vancomycin dosing intensity used was mg/kg/day. Prior MRSA infections were excluded due to the likely prior receipt of vancomycin.

### Definitions

The study team agreed upon the following definitions while designing this study. Nephrotoxicity was defined as an increase in serum creatinine (SCr) by greater than a 0.5 mg/dl or 50% increase from baseline on at least two consecutive laboratory tests during the period from initiation of vancomycin to completion of therapy [6]. We compared the nephrotoxicity rates among patients treated with guideline-recommended vancomycin doses (at least 30 mg/kg/day; at least 15 mg/kg/day for CrCl 30-50 ml/min) to those treated with lower vancomycin doses (less than 30 mg/kg/day; less than 15 mg/kg/day for CrCl 30-50 ml/min). Vancomycin trough concentrations were determined by the assay used for routine patient care at each institution. All concentrations labeled as “trough” were utilized. Only the first/initial vancomycin trough concentration was utilized in the analysis. All study hospitals did not have a mandatory therapeutic drug monitoring service and therefore vancomycin trough concentrations were only obtained as clinically indicated by the prescribing physician. Pitt bacteremia score and Charlson comorbidity index were used to quantify severity of illness and comorbid conditions. Both of these indices have been described in detail elsewhere [13-15].

### Statistical analysis

All analyses were performed using SAS 9.2 (Cary, North Carolina) and RTREE (Available at: http://c2s2.yale.edu/software/rtree/). It was determined that seven to eight variables would likely be evaluated in the multivariable model. In order to prevent overfitting, a total of 70-80 events would be required. Assuming a nephrotoxicity rate of 25% based on prior literature, 280-320 patients would be required for the multivariable analysis [1,7]. Candidate variables selected for consideration in the multivariable model were identified *a priori*. Univariable associations were explored using either Chi-square or Fisher’s Exact tests. Dichotomization of continuous variables was achieved by recursive partitioning to determine significant cut-points [16]. A Pitt bacteremia score ≥ 4 was used based on previous literature [17]. A vancomycin trough greater than 20 mcg/ml was based on previous observations of increased rates of nephrotoxicity compared with troughs of 15-20 mcg/ml [10]. The univariable analysis included the following candidate variables: receipt of guideline-recommended, weight-based vancomycin dosing, vancomycin trough greater than 20 mcg/ml, duration of vancomycin treatment greater than 15 days, gender, age greater than 52 years, weight greater than 100 kg, Pitt bacteremia score of 4 or higher, intensive care unit (ICU) residence, use of concomitant nephrotoxins (e.g. contrast dye, aminoglycosides, vasopressors), baseline serum creatinine, and Charlson comorbidity index score of 5 or higher.

Consideration for inclusion in the multivariable model was based on our conceptual model as well as significant associations observed in the univariable analysis (p<0.1). Independent predictors of nephrotoxicity were determined using a multivariable generalized linear mixed-effect model. Hospital site was treated as a random effect whereas other covariates were treated as fixed effects. A p value of <0.05 was considered statistically significant for the multivariable model. The analysis also included an extensive evaluation of effect measure modification and biologic interaction.

## Results

Of the 798 patients with MRSA bacteremia, 337 were included in the cohort (Hospital A = 156, Hospital B = 100, Hospital C = 81). Reasons for patient exclusion were not collected by the automated screening process. The baseline characteristics of the cohort are shown in Table 1. The cohort was predominantly male (79.2%) and was comprised of Caucasians (65%), African-Americans (14%), and Hispanics (17%). Data regarding race/ethnicity were missing for 5 patients and documented as other in seven patients. Vancomycin was dosed according to 2009 guidelines in 33.6% percent of patients. Patients weighing ≥ 100 kg received similar doses per day to those weighing < 100 kg (1941 mg vs. 1919 mg, p = 0.72). Patients receiving guideline-recommended, weight-based vancomycin dosing had a median daily dose of 32.0 mg/kg/day (interquartile range 29.0, 36.0), while those receiving lower doses received 21.3 mg/kg/day (interquartile range 17.0, 26.0) (p < 0.001). As expected, vancomycin trough concentrations were higher in patients receiving guideline-recommended, weight-based dosing (12.3 mcg/ml, IQR 8.3, 17.5) compared to patients who received lower doses (10.1 mcg/ml, IQR 7.1, 14.9), p = 0.03. The most common dosing frequencies administered were once (11.3%) or twice daily (86.3%). Other dosing frequencies included thrice daily (2.1%) or every other day (0.3%). The most common sources of infection for patients receiving weight-based vancomycin dosing according to the 2009 guideline were bloodstream catheter-related (16.6%), central nervous system (0.4%), gastrointestinal (0.9%), genitourinary (6.7%), osteomyelitis (1.3%), pulmonary (19.3%), skin/soft tissue (37.7%), and other (0.5%). The source of infection was undocumented for 16.6% of patients. For patients receiving lower doses, the sources of infection were bloodstream catheter-related (27.4%), genitourinary (10.6%), osteomyelitis (0.9%), pulmonary (15%), skin/soft tissue (20.4%). The source of infection was undocumented in 25.7% of patients receiving lower dosing.

**Table 1:** **Baseline characteristics of the cohort**^**A**^

**Characteristic**	Guideline-recommended dosing (n = 113)^*^	**Lower dosing (n = 223)**^**+**^	**p-value**
Male gender (%)	76%	81%	0.97
Age (years)	57 (46, 71)	54 (44, 63)	0.03
Height (cm)	172 (163, 178)	175 (168, 183)	<0.001
Weight (kg)	63 (57, 68)	88 (75, 104)	<0.001
Serum creatinine (mg/dl)	0.9 (0.7, 1.3)	0.9 (0.7, 1.2)	0.70
Creatinine clearance (ml/min)	71 (47, 104)	86 (66, 124)	<0.001
Charlson comorbidity index > 5	20%	13%	0.06
Length of hospital stay (days)	17 (9, 37)	18 (9, 32)	0.60
Intensive care unit resident	41%	41%	0.99
Pitt bacteremia ≥ 4	21%	22%	0.80
Concomitant nephrotoxin	50%	49%	0.85
Nephrotoxicity	22%	24%	0.74
Initial Vancomycin Dose (mg/kg/day)	32 (29, 36)	21 (17, 26)	<0.001

A = Results are presented as median (interquartile range) unless otherwise noted.

* = Vancomycin ≥ 30 mg/kg/day, ≥ 15 mg/kg/day for creatinine clearance of 30-50 ml/min.

+ = Vancomycin < 30 mg/kg/day, < 15 mg/kg/day for creatinine clearance of 30-50 ml/min.

Nephrotoxicity occurred in 78 patients (23%), occurring in 56%, 11%, and 33% of patients at Hospitals A, B, and C, respectively. The median (interquartile range) increase from baseline to peak serum creatinine was 0.0 mg/dL (0.0, 0.2) for patients who did not develop nephrotoxicity versus 1.0 mg/dL (0.6, 2.1) for patients who developed nephrotoxicity. Fifteen percent of patients had a vancomycin trough concentration greater than 20 mcg/ml. Concurrent nephrotoxins included contrast dye (34%), aminoglycosides (19%), and vasopressors (12%). Concomitant antimicrobials active against MRSA were used in 23% of patients.

In the univariable analysis (Table 2), nephrotoxicity was similar between patients that received guideline-recommended, weight-based vancomycin dosing versus lower dosing (22% vs. 24%). Factors associated with increased risk for nephrotoxicity included duration of vancomycin treatment greater than 15 days, weight greater than 100 kg, Pitt bacteremia score of 4 or higher, vancomycin trough greater than 20 mcg/ml, age greater than 52 years, ICU residence, and concomitant nephrotoxin. In the multivariable analysis there was not a statistically significant association between vancomycin dosing and nephrotoxicity (Table 3). Independent predictors of nephrotoxicity in the multivariable model were duration of vancomycin treatment greater than 15 days, weight greater than 100 kg, Pitt bacteremia score of 4 or higher, vancomycin trough greater than 20 mcg/ml, and age greater than 52 years.

**Table 2:** Univariable analysis of risk factors for nephrotoxicity

**Variable**	**Odds ratio**	**95%confidence interval**
Guideline-recommended vancomycin dosing	0.91	0.53-1.56
Vancomycin duration > 15 days	3.65	2.16-6.17
Weight greater than 100 kilograms	2.08	1.18-3.67
Pitt Bacteremia Score of four or greater	3.80	2.17-6.63
Vancomycin trough greater than 20 mcg/ml	2.51	1.28-4.92
Age greater than 52 years	2.40	1.38-4.15
Intensive care unit resident	3.64	2.15-6.18
Concomitant nephrotoxin	2.08	1.24-3.49
Male gender	1.04	0.56-1.57
Charlson comorbidity index 5 or greater	1.14	0.57-2.27
Baseline serum creatinine greater than 1.0 mg/dL	0.94	0.56-1.57

**Table 3:** Multivariable analysis of independent risk factors for nephrotoxicity

**Variable**	**Odds ratio**	**95%confidence interval**
Guideline-Recommended Vancomycin Dosing	1.52	0.75-3.08
Vancomycin duration greater than 15 days	3.36	1.79-6.34
Weight greater than 100 kilograms	2.74	1.27-5.91
Pitt Bacteremia Score of four or greater	2.73	1.29-5.79
Vancomycin trough greater than 20 mcg/ml	2.36	1.07-5.20
Age greater than 52 years	2.10	1.08-4.08
Intensive care unit residence	1.90	0.95-3.80
Concomitant nephrotoxin	1.64	0.87-3.11

## Discussion

We did not observe a statistically significant relationship between the receipt of guideline-recommended, weight-based dosing of vancomycin and the development of nephrotoxicity in patients in our cohort. This finding is clinically important because weight-based vancomycin dosing is now recommended by the vancomycin guidelines [6].

The impact of vancomycin on the development of nephrotoxicity has been debated for decades. Multiple prospective clinical trials suggest that traditional vancomycin doses (1 gram IV every 12 hours) cause nephrotoxicity 5% of the time, or less, when concomitant nephrotoxins are not used [18-21]. A 7-35% rate of nephrotoxicity was reported with the concomitant use of nephrotoxins [22,23].

The controversy surrounding vancomycin-associated nephrotoxicity has resurfaced in parallel with the increased utilization of higher vancomycin doses to achieve higher target trough concentrations in response to rising vancomycin MIC values. The nephrotoxicity rate in our study (23%) is consistent with recent data utilizing a standard definition of nephrotoxicity (11-42%) [1,7-10].

Our study also agreed with other studies that have identified an association between vancomycin trough concentrations and nephrotoxicity [1,7-10]. These studies, and our own, are unable to determine whether this association is causative in nature. Our observation of an association between duration of vancomycin treatment and nephrotoxicity is also consistent with previous work [1,7,9]. Whether vancomycin duration is a causative factor in nephrotoxicity is also unclear.

The factors significantly associated with the development of nephrotoxicity in our study are clinically reasonable. We found that a Pitt bacteremia score of 4 or greater was predictive of nephrotoxicity in our study. This is consistent with other studies that have observed a significant association between nephrotoxicity and ICU residence upon antibiotic initiation or increased APACHE II scores [7,8,24]. We observed that greater patient weight was significantly associated with the development of nephrotoxicity, which is also consistent with previous investigations [8,24].

The application of our study is limited by its retrospective nature and the potential lack of external validity in patients being treated with vancomycin for conditions other than MRSA bacteremia. The lack of information regarding reasons for exclusion may also limit how other institutions are able to use these findings for their patient population. Retrospective studies may have differences between the comparison groups in regards to measured and unmeasured confounders. To address potential confounding, a stratified analysis was conducted on variables considered to potentially affect the primary outcome. Biologically plausible factors that demonstrated confounding were included in the multivariable model. A multivariable mixed-effects model using hospital site as the random effect was utilized to minimize the impact of the differences in measured confounders as well as the risk of clustering. One major difference between the institutions is the much longer length of stay at Hosptial A due to two long-term care wings in the facility compared to none for the Hospitals B and C. Therefore, the higher nephrotoxicity rate at Hospital A may be in part due to an observation bias due to patients on vancomycin remaining in the hospital longer than patients at the other institutions.

The fact that we did not observe an association between concomitant nephrotoxins and nephrotoxicity may have been due to the lack of recording the dose and duration of concomitatnt nephrotoxin use. Our results may have also been subject to a selection bias since patients weighing greater than 70 kilograms were less likely to receive weight-based vancomycin dosing as recommended by the 2009 guideline. This selection bias could have reduced our ability to detect guideline-recommended, weight-based dosing as an independent risk factor for nephrotoxicity. The utilization of all vancomycin concentrations labeled as “troughs” could have biased our results. The potential for each institution to use different vancomycin assays could have also biased our results. However, our study found that vancomycin troughs greater than 20 mcg/ml is associated with nephrotoxicity mirroring those of previous studies. This lack of effect is common for most non-differential misclassification biases. If any effect were to occur to this bias, it would have been to lessen the ability to determine that vancomycin trough concentrations are a risk factor for nephrotoxicity. The exclusion of patients with missing vancomycin trough concentrations from the multivariable model may have also biased the results. This exclusion may have created a selection bias that decreased the ability to detect severity of illness or length of vancomycin therapy as patients without therapeutic drug monitoring tend to be less severely ill patients who do not require long durations of therapy. The fact that both of these characteristics remained independent predictors of nephrotoxicity in spite of this selection bias reinforces the strength of these associations. Last, our study evaluated dosing practices prior to the publication of the 2009 guidelines. However, this standard measure created two distinct groups supported by the 2009 guideline regardless of how often weight-based vancomycin was utilized during the study period.

Furthermore, the implications of loading doses and therapeutic drug monitoring programs on nephrotoxicity need further evaluation since none of our institutions utilized loading doses or formal therapeutic drug monitoring services (e.g. automatic pharmacy consultation for vancomycin management). Each study institution employs clinical pharmacists who monitor vancomycin trough concentrations and provide recommendations for the physician to clinically evaluate.

## Conclusions

In this multi-center study, more than one in five patients developed nephrotoxicity. We did not observe a significant relationship between weight-based guideline-recommended dosing and nephrotoxicity. If this finding is confirmed by others, clinicians should be able to utilize weight-based, guideline-recommended dosing. Careful management of patients with MRSAB is needed to avoid vancomycin trough concentrations associated with nephrotoxicity.

## Competing interests

Grant funding from AstraZeneca, Ortho-McNeil Janssen, and Pfizer: CRF. Scientific Advisory Board for Tibotec Therapeutics and Gilead Sciences: RJB. None: RGH, CAA, CAG, KKH, KAH, NAF, SDB, TB.

## Author contributions

RGH, CAG, KKH, KAH, SDB, RJB were involved in the study concept and design. RGH, CAG, CAA, CRF were involved in the data analysis and interpretation. RGH, CAG, KKH, KAH, CAA, CRF, NAF, SDB, TB, RJB were involved in the drafting of the manuscript for important intellectual content and had final approval of the manuscript.

## Pre-publication history

The pre-publication history for this paper can be accessed here:

http://www.biomedcentral.com/2050-6511/14/12/prepub

## References

[B1] Hidayat LK Hsu DI Quist R Shriner KA Wong-Beringer A High-dose vancomycin therapy for methicillin-resistant Staphylococcus aureus infections: efficacy and toxicity Arch Intern Med 2006 166 2138 2144 10.1001/archinte.166.19.2138 17060545

[B2] Lodise TP Graves J Evans A Relationship between vancomycin MIC and failure among patients with methicillin-resistant Staphylococcus aureus bacteremia treated with vancomycin Antimicrob Agents Chemother 2008 52 3315 3320 10.1128/AAC.00113-08 18591266 PMC2533486

[B3] Sakoulas G Moise-Broder PA Schentag J Forrest A Moellering RC Jr Eliopoulos GM Relationship of MIC and bactericidal activity to efficacy of vancomycin for treatment of methicillin-resistant Staphylococcus aureus bacteremia J Clin Microbiol 2004 42 2398 2402 10.1128/JCM.42.6.2398-2402.2004 15184410 PMC427878

[B4] Wang G Hindler JF Ward KW Bruckner DA Increased vancomycin MICs for Staphylococcus aureus clinical isolates from a university hospital during a 5-year period J Clin Microbiol 2006 44 3883 3886 10.1128/JCM.01388-06 16957043 PMC1698298

[B5] Tenover FC Moellering RC Jr The rationale for revising the clinical and laboratory standards institute vancomycin minimal inhibitory concentration interpretive criteria for staphylococcus aureus Clin Infect Dis 2007 44 1208 1215 10.1086/513203 17407040

[B6] Rybak M Lomaestro B Rotschafer JC Therapeutic monitoring of vancomycin in adult patients: a consensus review of the American Society of Health-System Pharmacists, the Infectious Diseases Society of America, and the Society of Infectious Diseases Pharmacists Am J Health Syst Pharm 2009 66 82 98 10.2146/ajhp080434 19106348

[B7] Jeffres MN Isakow W Doherty JA Micek ST Kollef MH A retrospective analysis of possible renal toxicity associated with vancomycin in patients with health care-associated methicillin-resistant Staphylococcus aureus pneumonia Clin Ther 2007 29 1107 1115 10.1016/j.clinthera.2007.06.014 17692725

[B8] Lodise TP Patel N Lomaestro BM Rodvold KA Drusano GL Relationship between initial vancomycin concentration-time profile and nephrotoxicity among hospitalized patients Clin Infect Dis 2009 49 507 514 10.1086/600884 19586413

[B9] Pritchard L Baker C Leggett J Sehdev P Brown A Bayley KB Increasing vancomycin serum trough concentrations and incidence of nephrotoxicity Am J Med 2010 123 1143 1149 10.1016/j.amjmed.2010.07.025 21183005

[B10] Kullar R Davis SL Levine DP Rybak MJ Impact of vancomycin exposure on outcomes in patients with methicillin-resistant staphylococcus aureus bacteremia: support for consensus guidelines suggested targets Clin Infect Dis 2011 52 975 981 10.1093/cid/cir124 21460309

[B11] Hall RG Giuliano CA Haase KK Empiric guideline-recommended weight based dosing and mortality in methcillin-resistant Staphylococcus aureus bacteremia: a retrospective cohort study BMC Infect Dis 2012 12 104 10.1186/1471-2334-12-104 22540223 PMC3532187

[B12] Cockcroft DW Gault MH Prediction of creatinine clearance from serum creatinine Nephron 1976 16 31 41 10.1159/000180580 1244564

[B13] Charlson ME Pompei P Ales KL MacKenzie CR A new method of classifying prognostic comorbidity in longitudinal studies: development and validation J Chronic Dis 1987 40 373 383 10.1016/0021-9681(87)90171-8 3558716

[B14] Chow JW Fine MJ Shlaes DM Enterobacter bacteremia: clinical features and emergence of antibiotic resistance during therapy Ann Intern Med 1991 115 585 590 1892329 10.7326/0003-4819-115-8-585

[B15] Chow JW Yu VL Combination antibiotic therapy versus monotherapy for gram-negative bacteraemia: a commentary Int J Antimicrob Agents 1999 11 7 12 10.1016/S0924-8579(98)00060-0 10075272

[B16] Zhang H Singer B Recursive partitioning in the health sciences 1999 New York: Springer

[B17] Rhee JY Kwon KT Ki HK Scoring systems for prediction of mortality in patients with intensive care unit-acquired sepsis: a comparison of the Pitt bacteremia score and the Acute Physiology and Chronic Health Evaluation II scoring systems Shock 2009 31 146 150 10.1097/SHK.0b013e318182f98f 18636041

[B18] Arbeit RD Maki D Tally FP Campanaro E Eisenstein BI The safety and efficacy of daptomycin for the treatment of complicated skin and skin-structure infections Clin Infect Dis 2004 38 1673 1681 10.1086/420818 15227611

[B19] Ellis-Grosse EJ Babinchak T Dartois N Rose G Loh E The efficacy and safety of tigecycline in the treatment of skin and skin-structure infections: results of 2 double-blind phase 3 comparison studies with vancomycin-aztreonam Clin Infect Dis 2005 41 S341 S353 10.1086/431675 16080072

[B20] Weigelt J Itani K Stevens D Lau W Dryden M Knirsch C Linezolid versus vancomycin in treatment of complicated skin and soft tissue infections Antimicrob Agents Chemother 2005 49 2260 2266 10.1128/AAC.49.6.2260-2266.2005 15917519 PMC1140485

[B21] Wilcox MH Tack KJ Bouza E Complicated skin and skin-structure infections and catheter-related bloodstream infections: noninferiority of linezolid in a phase 3 study Clin Infect Dis 2009 48 203 212 10.1086/595686 19072714

[B22] Downs NJ Neihart RE Dolezal JM Hodges GR Mild nephrotoxicity associated with vancomycin use Arch Intern Med 1989 149 1777 1781 10.1001/archinte.1989.00390080053013 2764651

[B23] Sorrell TC Collignon PJ A prospective study of adverse reactions associated with vancomycin therapy J Antimicrob Chemother 1985 16 235 241 10.1093/jac/16.2.235 3934126

[B24] Lodise TP Lomaestro B Graves J Drusano GL Larger vancomycin doses (at least four grams per day) are associated with an increased incidence of nephrotoxicity Antimicrob Agents Chemother 2008 52 1330 1336 10.1128/AAC.01602-07 18227177 PMC2292536

